# Using Neural Networks in Order to Analyze Telework Adaptability across the European Union Countries: A Case Study of the Most Relevant Scenarios to Occur in Romania

**DOI:** 10.3390/ijerph182010586

**Published:** 2021-10-09

**Authors:** Ana Maria Mihaela Iordache, Codruța Cornelia Dura, Cristina Coculescu, Claudia Isac, Ana Preda

**Affiliations:** 1 Department of Informatics, Statistics and Mathematics, Romanian–American University, 012101 Bucharest, Romania; coculescu.cristina@profesor.rau.ro; 2 Department of Economics, University of Petroșani, 332006 Petroșani, Romania; claudiaisac@upet.ro (C.I.); ssu@upet.ro (A.P.)

**Keywords:** telework adaptability, cluster analysis, classification, remote work, neural network, perceptron, SAS Enterprise Miner, SAS Enterprise Guide

## Abstract

Our study addresses the issue of telework adoption by countries in the European Union and draws up a few feasible scenarios aimed at improving telework’s degree of adaptability in Romania. We employed the dataset from the 2020 Eurofound survey on Living, Working and COVID-19 (Round 2) in order to extract ten relevant determinants of teleworking on the basis of 24,123 valid answers provided by respondents aged 18 and over: the availability of work equipment; the degree of satisfaction with the experience of working from home; the risks related to potential contamination with SARS-CoV-2 virus; the employees’ openness to adhering to working-from-home patterns; the possibility of maintaining work–life balance objectives while teleworking; the level of satisfaction on the amount and the quality of work submitted, etc. Our methodology entailed the employment of SAS Enterprise Guide software to perform a cluster analysis resulting in a preliminary classification of the EU countries with respect to the degree that they have been able to adapt to telework. Further on, in order to refine this taxonomy, a multilayer perceptron neural network with ten input variables in the initial layer, six neurons in the intermediate layer, and three neurons in the final layer was successfully trained. The results of our research demonstrate the existence of significant disparities in terms of telework adaptability, such as: low to moderate levels of adaptability (detected in countries such as Greece, Croatia, Portugal, Spain, Lithuania, Latvia, Poland, Italy); fair levels of adaptability (encountered in France, Slovakia, the Czech Republic, Hungary, Slovenia, or Romania); and high levels of adaptability (exhibited by intensely digitalized economies such Denmark, Sweden, Finland, Germany, Ireland, the Netherlands, Belgium, etc.).

## 1. Introduction

The accelerated spread of the COVID-19 pandemic around the globe at the beginning of 2020 has shaken up the deepest roots of our social systems and has stood out as an extraordinary challenge, especially within the areas of public health, economy, and education [1]. People have had to adapt to new restrictions and have tried to conduct most of their gainful activities while maintaining social distancing in order to avoid the spread of the infection of the new virus. Thus, the exchanges of tangible or intangible resources through alternative channels have been tremendously intensified. 

If remote work had already seen a modest development over the past few years, this tendency was reversed during the pandemic, and telework became an almost normal situation across the continent. The starting point of our research was represented by the release of the results of a questionnaire conducted by Eurofound at the EU level on the issue of remote work; the study inspired us to deepen the analysis on working conditions during the pandemic in order to extract information that was less visible than the naked eye [2]. Our investigations were also instrumental in developing some recommendations on increasing employee willingness in adopting new remote work patterns. 

Therefore, we performed an explorative study to precisely investigate three interconnected research questions (RQs):

1. How can EU countries be classified in relation to the degree of employee telework adoption during the emergency measures adopted by governmental authorities in 2020?
2. Which are the fundamental features that can be highlighted for the countries included in each class and what kind of recommendations can be made in terms of increasing the adaptability to these new work arrangements?
3. Which are the main scenarios to be implemented by the decision makers from Romania in order to ensure the smooth transition of the country from a class with a moderate level of remote work implementation into a class with a higher ranking pertaining to telework adoption?

The reminder of the paper is organized as follows: Section 1 represents the introduction of the paper and emphasizes the high relevance of the topic of telework during the rapid proliferation of the SARS-CoV-2 virus. Section 2 is dedicated to the objective of establishing a conceptual framework for telework in order to offer a reference point for researchers and practitioners from the field; this part of our study has been organized into two distinct sub-sections:

✓ In paragraph 2.1, we took stock of the core concepts and debates from the literature regarding the astonishing evolution of telework alongside three overlapping generations: home office; mobile office; and virtual office;
✓ Paragraph 2.2 was devoted to a short presentation of the state of the art regarding the telework patterns implemented before and during the COVID-19 pandemic period; the main implications on the level of adoption propensity manifested both by employers and employees from the European Union were also discussed.

Section 3 offers insights into the research design and methods by introducing neural network applications and their potential to model and conceive efficient solutions for real-world problems that have emerged within the business sphere. Within this section, the authors focus on presenting the main steps followed by the process of training a neural network in order to analyze the degree of remote work embracement: the collection and preparation of initial data; the employment of cluster analysis; the basis of the Levenberg–Marquardt algorithm selected in order to provide an accurate answer to our research questions; the effective training of the neural network with the help of SAS Enterprise Miner software; the forecasting scenarios performed for the Romanian case, etc. 

Section 4 deals with the presentation of our empirical results: the descriptive statistics for the research variables; the cluster analysis; the evolution of the training errors and calculations performed in order to validate the neural network model; the estimation of the weights from our input data to neurons that form the hidden layer; the values of transfer functions; and the three main groups of countries individualized according to their degree of adaptability to teleworking. 

Section 5 provides a comparison of our findings with other relevant results highlighted by recent studies approaching the same topic. The most relevant scenarios to occur in Romania regarding the possibility of increasing the level of remote work embracement during the COVID 19 pandemic have been given extensive coverage within this section. 

Finally, Section 6 ends our study by drawing a few relevant conclusions and policy implications generated by the inevitable upsurge trend in telework adoption in the post-COVID era. The inherent limitations and the future challenges and avenues of research are also addressed within this part of our paper.

## 2. Conceptual Framework of Telework and Literature Review

### 2.1. The Conceptual Framework

The concept of telework originated in the early 1970s to designate a new procedure of implementing unconventional work settlements. The locution “telecommuting network” was coined by Jack M. Niles and denotes the removal of daily travel to work due to the proliferation of ICT usage. [3]. Despite the fact that a widely acknowledged definition of the phenomenon has not been determined yet, the term telework is commonly recognized as the equivalent to any work arrangement that is performed outside the employer’s premises and implies up-to-date computer technology usage. 

Consequently, telework can be performed from numerous types of places (home, office, and other different locations) on the grounds of various types of technologies and with different periodicity. An interesting concretization of various modes to telework implementation came from the ”Statistical Indicators Benchmarking the Information Society (SIBIS)”, who described four distinct telework modes: telework performed from home, mobile telework, freelance telework in small offices/home offices, and telework accomplished in collaborative facilities, external from institutions and employee residences [4].

As we can see, the telework concept is embedded in the literature and is in accordance with the historical evolution of ICTs and the corresponding work arrangements. Thus, research belonging to the “traditional generation” of telework was focused on a unique procedure of putting new work arrangements into effect—the Home Office—as computers and other electronic devices developed at that time were unable to facilitate employee flexibility during their working schedule. However, over the last few years, the occurrence of highly adjustable “cloud-based” working places, accessible by virtue of smartphones, laptops, and tablets from basically any spot on earth, can make the concept of telework (as initially defined) appear rather anachronistic [5,6]. 

Moreover, it is quite difficult to distinguish the first telework generation from the second—the Mobile Office—due to the fact that progress took place step-by-step at different paces and with different nuances throughout institutions, branches, and countries. Its smooth evolution was mainly driven by two determinants: (i) the increasing pace of technology development; (ii) substantial changes in government regulations, which were made in order to align teleworkers to the same requirements that traditional workers had to fulfill in relation to their employers. As far as organizational workspaces are concerned, the so-called “third spaces” (situated between home and work) are typical for the second generation: co-working areas, innovation hubs, open workshops, living labs, start-up incubators, etc. [7]. Among the most evident advantages brought about by this working structure, we can mention the following: greater opportunities to access high-level technologies; low intensity isolation risks; diminishing trends of personnel expenses for employers (determined by low commuting costs or by delivering permission for employees to use inexpensive facilities); an enhanced sense of achievement regarding one’s job and well-being, etc. [8]. 

The seeds of the third telework generation—the Virtual Office—can already be detected since the World Wide Web has undergone a series of transformations to enable higher flexibility, accessibility, and interoperability for users from all over the Globe [9]. From a technological point of view, there have been some relevant predictions in the literature that have painted a complex picture for the future: access to high-speed Internet through radio waves in conjunction with downsized transistors would determine the adoption of new ICTs, i.e., miniature devices (such as smartphones and other similar instruments) and would be able to access a large amount of information that has already preserved within clouds and networks [10]. Although this prognosis was made back in 1997, it is true nowadays, and it illustrates the real possibility of an employee to attend work away from their employer’s premises, anywhere and anytime [11]. Thus, the third telework generation will entail smart or agile working, a procedure of pursuing everyday job obligations partially from the office and partially in a remote manner in order to meet the needs of employees who are seeking to harmonize their professional life with family responsibilities. As far as the workplace is concerned, smart working simply empowers the employee to freely choose the location as a substitute for the organization’s premises, which may be his/her own residence, in parallel with a cooperative space or a public library or other major interest institution [8]. 

**Figure 1:**
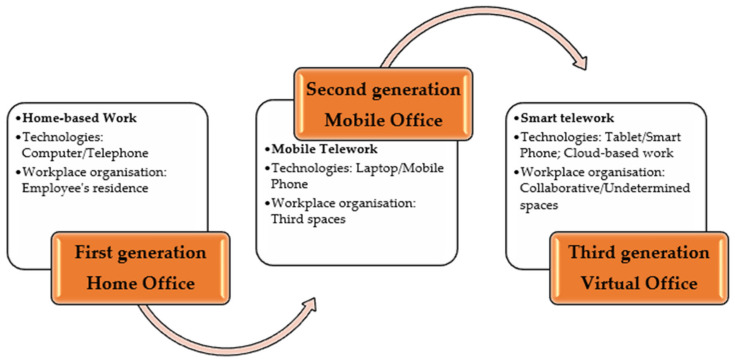
The conceptual framework for telework [9].

Starting from this evolutionary perspective on telework emergence, we have constructed a conceptual framework of telework, which is adapted from John Messenger’s forward-looking approach, introduced in the book “Telework in the 21st Century” (see Figure 1). 

Surprisingly, some authors have argued that the rapid proliferation of cheap and productive ICT devices alone was not able to foster high levels of telework adoption before the pandemic. Thus, other determinants such as personal views on job appropriateness, prestige or authority issues, the employers’ willingness to apply new work arrangements, the peculiarities of the organizational culture, etc., appeared to have been as equally important as the technological developments [3]. However, most of the impediments that used to block telework adoption before COVID 19 were removed in the beginning of 2020, when governmental authorities from all over the world encouraged companies to promote telework in order to avoid workplace overcrowding. 

In the next paragraph, we shall perform a state-of-the-art analysis focused on the direct or subsequent effects induced by telework while maintaining an evolutionary-based perspective regarding different aspects involved in remote work implementation (such as work modes, ICTs tools, and working spaces formulae). Moreover, we shall lay stress the massive acceleration of telework adoption by EU countries during the COVID 19 outbreak in order to prepare the groundwork for the process of assessing certain expected future scenarios in this area. 

### 2.2. Teleworking in the EU in Times of COVID-19 Pandemic—A Short Literature Review

Following the tremendous technological advancements highlighted in the above, the debates on telework issues continue to receive a great deal of attention in the literature, the main emphasis being laid over the last two years, specifically on the ways in which this new paradigm was put into practice under the circumstances of the crisis associated with the propagation of COVID 19 disease. 

Prior to the outbreak of the pandemic, the prevalence rate of teleworking across EU countries was quite low. Thus, according to the 2018 European Labor Force Survey, the share of individuals who worked from home on a regular basis represented only 5% of employees, while another share of 10% was illustrated by part-time teleworkers. The percentages provided by the survey were converted into an indicator that varies between 0 (for the economic sectors that exclude telework) and 100 (for the sectors that involve the entire personnel in the process of teleworking)—see Table A1 [12]. The general value determined for this indicator at the European Union level was 10.24, and it has been used as a relevant basis for comparisons between sectors and countries; the highest values were registered within the economic domains categorized as “teleworkable” (i.e., those sectors that display a high level of material capability for supporting remote work arrangements) [13]. 

However, even in the most teleworkable domains (such as services, ICTs, education, public administration, and defense, etc.) the approximated telework prevalence was rather low before the crisis—approximately 17.5. As a result of examining the statistical data from Table A1, we must emphasize the important differences among the same type of sectors from different countries. For example, even in the lower-ranking teleworkable sectors, the Northern countries in the EU have larger fractions of teleworkers than those registered by Italy, Portugal, and Spain in their most teleworkable sectors [12]. Another study conducted in the first part of 2020 [14] showed that the effects generated by the introduction of remote work vary to a large extent between economic sectors. Thus, in the case of countries and companies that had adopted telework before (Northern European countries), the transition was smoother, compared to other countries (Central and Southern European countries) [15]. Whether they are clients or employees in various sectors of activity, time has shown that young people or those who are already accustomed to this type of activity are the ones who adapt the most easily to distance work [16].

Nevertheless, in 2020, in the context of the unfolding of the health crisis, employees who could accomplish their job duties from home were encouraged to proceed in that manner. The pandemic has behaved as a driver for telework expansion [17]. Consequently, as a result of substantial changes in working the conditions conducted by European governments during the emergency state, 37% of the labor force began to work from home [3]. As expected, the activities that were the most affected by the COVID-19 pandemic were those in which direct human interaction was absolutely necessary (the so-called “non-teleworkable” sectors). For example, libraries needed to adapt to working in the cloud environment in order to provide access to remote readers [18].

**Figure 2:**
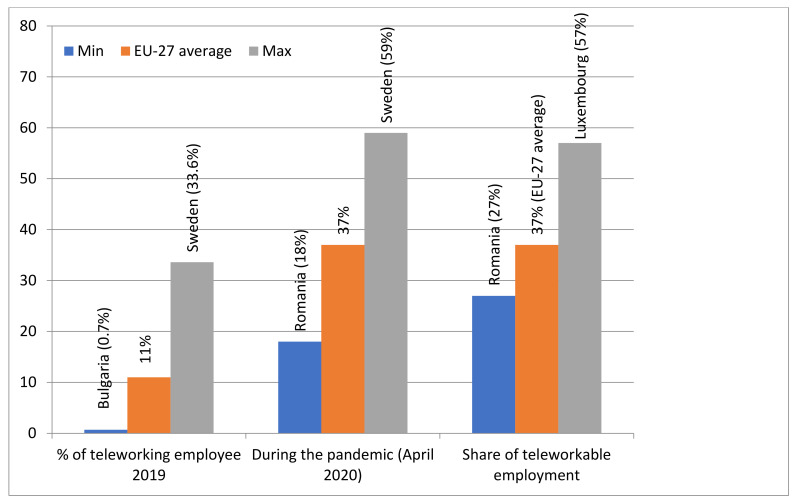
Changing the percentage of employees who use telework before and during the Covid-19 pandemic. Source: Authors’ elaboration from [13].

As shown in Figure 2, according to the Eurofound dataset, the share of teleworking employees during the COVD-19 crisis (April 2020) revealed significant differences between the EU27 countries, ranging between 18% in Romania and 59% in Sweden. Moreover, the share of the workforce in teleworkable occupations fluctuates between 35% and 41% for two thirds of the EU27 countries, with a peak in Luxembourg (54%) and the lowest value in Romania (27%) [13].

Over the last two years, the effects brought about by remote work at various levels of society have been at the forefront of scholarly debates. Hence, a recent study shows the possibility of outsourcing certain activities, using remote work, from the most expensive housing markets of the United States to the cheapest ones [19]. Several major benefits—such as higher income for employees, lower labor costs for companies, the complete cutting down of employee commute time, etc.—derive from the accelerated rhythm of telework implementation. By conducting interviews with companies that have adopted telework, another study approaching a new work-from-home model emphasizes that since daily travel to work is no longer necessary, managers can hire talented people from various parts of the Globe [20]. However, at the local level, the implications are also remarkable, from the processes of dissolving companies (while adapting employees to peculiarities of telework) to fueling economic development supported by opportunities brought about by globalization and ITC advancements [21,22].

On the other hand, some authors have argued that the COVID-19 pandemic was able to accelerate a phenomenon that would have materialized in any case; nevertheless, the sanitary crisis increased the disparities between the traditional and the digital economy, the excellent and the lowest-performing companies, the educated and uneducated employees, prosperous and underprivileged people, even at the country level [23,24].

Thus, a significant difference between compulsory teleworking brought about by the crisis and remote work in normal conditions has been illustrated in the literature. A comparative study performed in companies from Poland, Lithuania, and Spain showed that pandemic-induced telework caused exacerbated levels of stress and anxiety among employees, as their organizational structures and technological capabilities were little prepared to confront a high-magnitude transmutation such as the rapid adoption of remote work [25]. On the same train of thought, entrepreneurial features such as high risk appetite, creativity, the ability to implement new digital workplace strategies, and the ability to go over the unprecedented opportunities that emerged in marketplaces have been pointed out as key-success factors that have made a difference in terms of company survival in times of crisis. For this purpose, we consider that there is a need for future studies regarding the characteristics of the entrepreneurial work environment in relation to the pandemic, as these studies would offer original and efficient solutions to address challenges called forth by the crisis [26]. 

Although the roots of these new working arrangements were present before the pandemic, the abrupt shift to teleworking brought about a number of significant drawbacks, particularly for the most vulnerable people either due to the low degree of teleworkability of their occupation or due to other personal factors such as age, level of education, IT skills, or psychological impacts induced by the challenges or remote work [23,24,27,28].

Thus, there is no common consent in the literature regarding the worldwide upsurge of the telework phenomenon during the pandemic. Early research brought into to view that the majority of the companies and employees consider that the “overnight “adoption of teleworking neglected the requirements towards a certain degree of readiness in terms of rearranging work-from-home settings in order to consolidate job satisfaction parameters [25]. In line with this hypothesis, two independent studies performed in Romania before and during the pandemic showed that the organizational environment represents a determinative factor in adjusting the multifaceted aspects of job satisfaction: the level of autonomy in performing tasks; the flexibility of the work schedule; the employee work–life balance; social interactions with colleagues and costumers from outside of the company; work design characteristics, etc. If the first-mentioned drivers could enhance the level of job satisfaction for remote workers, the others might exert a negative impact upon it. 

This mixed landscape of documentation regarding the remote working phenomenon needs to be complemented by a comprehensive analysis of personal factors that undoubtedly impact job satisfaction level, such as an employee’s gender, education, age, the proactive attitude towards changes, the number of dependent children, the marital status, the quality of the working space available at home, etc. [28,29]. In other words, the expansion of the work-from-home phenomenon caused by the global pandemic crisis still represents an under-researched issue with miscellaneous evidence of both positive and negative implications.

Looking ahead to the post COVID era, both employees and institutions must make a crucial choice regarding the extent to which they wish to perpetuate telework patterns. Thus, organizations that promote remote work need to make the extensive use of the cloud environment, have to have sufficiently developed servers to store enormous amounts of information, and, most importantly, they have to pay due attention to data security [30].

Despite the comfort felt by some remote employees, specialists recommend a high level of compliance with certain rules related to ergonomics and time management [31,32]. For instance, companies such as Twitter and Google offer their employees a full-time telework schedule. On the opposite side, in other organizations, not all employees work remotely, so there are situations where some employees may feel isolated, unsynchronized, and without perspective [33]. This is why the managers of the future must adopt to those work patterns that can keep employees motivated, productive, and with an optimum balance between personal and professional life, provided that human interaction will be no longer a necessary condition for the smooth running of things [34].

## 3. Research Methodology

### 3.1. The Neural Network Models—Theoretical Grounds

In economics, neural networks (NN) have produced, especially since 1990s, a large body of research through the medium of which new models have become deep-rooted as a valid alternative to conventional statistical models. The comprehensive scope of applications has covered different areas such as macroeconomic forecasts, bankruptcy predictions, stock market dynamics, credit evaluations, marketing segmentation, decision-making processes, etc. [35,36]. Due to their huge potential to determine and replicate nonlinear and linear associations amongst various groups of variables, artificial neural networks have emerged as widespread and efficient models that can be applied for classification reasons, clustering issues, pattern detection, and forecasting operations in many other fields that are connected to business [37]. 

The build-up process of neural networks originates from biology; thus, NN represent a simplified model of the human brain aimed at simulating the intelligence patterns of learning and storing large amounts of knowledge through tightly interconnected neurons [38,39]. Due to their flexibility, learning properties, and generalization power, an important number of NN architectures have been advanced in the literature. Among these categories, we selected a back-propagation learning algorithm with a feed-forward design. This model consists of artificial neurons organized in three different types of layers: the input layer, one or several hidden layers, and an output layer. 

**Figure 3:**
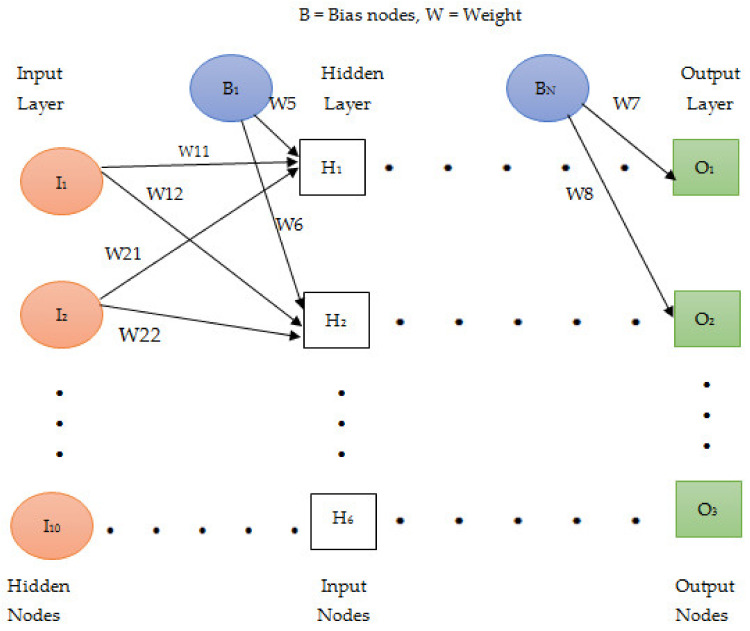
Arhitecture of an artificial neural network with one hidden layer, ten input nodes and three output nodes. Adapted from [38].

The neurons that make up a layer can be also called units. The units arranged in every single layer are linked to those operating in the neighboring layers (via respective weights and biases which are manifest for every single interrelation). Thus, the numerous weights that are contained within the network architecture correspond to the estimated coefficients of a classical regression model while the bias can simply be paralleled with the intercept term. Figure 3 shows the classical architecture of a feed-forward NN with one hidden layer.

The training of a NN implies that a certain amount of information (which constitutes the training dataset) is supplied into the network through the input units; it moves across the hidden layers and moves to the output units. Each unit has to reckon the weighted average of its inputs; the outcome calculated as the sum of the weighted averages for every single input is produced through a non-linear function. The unit starts running when the total value of the determined sum is higher than a previously defined threshold and, consequently, it activates the adjacent units from the next layer. Through this mode of action, a NN can establish complex non-linear associations between explanatory and response variables.

Moreover, a feedback dimension is incorporated in the process of NN functioning. Thus, the results provided by the network are set in opposition with the factual value expected to be generated; the difference between the two values is used in order to adjust the weights corresponding to the associations between units. The machine learning algorithm described here is acknowledged as “back propagation” [40].

The moment the learning process is completed, an unseen dataset known as the testing dataset is employed in order to assess the generalization power of the categorizer. Consequently, the validation dataset represents a group of data that is not at all involved in the process of NN training (or, in other words, the algorithm never “encounters” the validation data), but it acts as a marker that highlights the out-of-sample prediction rigorousness of the network.

Following each iteration performed in the assessment process, an out-of-sample prediction is drawn up by using the values that make up the data set, and the mean squared error (MSE) is determined. The network that minimizes MSE for the validation dataset indicates the most accurate alternative of the model. The process of training the neural network continues by repeatedly changing the neurons’ weights, and stops at the moment the output value reaches the expected target [41].

In order to develop an NN algorithm, we must start from the basic process, which is known as “single perceptron”. In other words, a perceptron amounts to a unique McCulloch–Pitts neuron, which works with adjustable weights and bias. In an attempt to build a multilayer perceptron, the perceptron is adapted in a manner that allows it to incorporate a few layers of neurons together with nonlinear activation functions that ensures higher model potency since it can be applied in cases of nonlinear independent datasets [42].

The following paragraph contains an in-depth presentation of the main stages included in our methodology, which were conceived in order to build a neural network for the purpose of classifying EU countries.

### 3.2. Research Design

As stated above, our analysis is aimed at taking into account the main tendencies of the teleworking upsurge within EU countries in the midst of the pandemic and to outline a few scenarios that could determine a significant increase in the implementation of these new work arrangements in Romania. In order to accomplish our goal, we have used data extracted from a survey on living and working issues during COVID-19, which were based on a questionnaire that was conducted online at the European Union level by Eurofound between 22 June–27 July 2020 (Round 2), with 24,123 complete responses provided by respondents aged 18+. 

**Figure 4:**
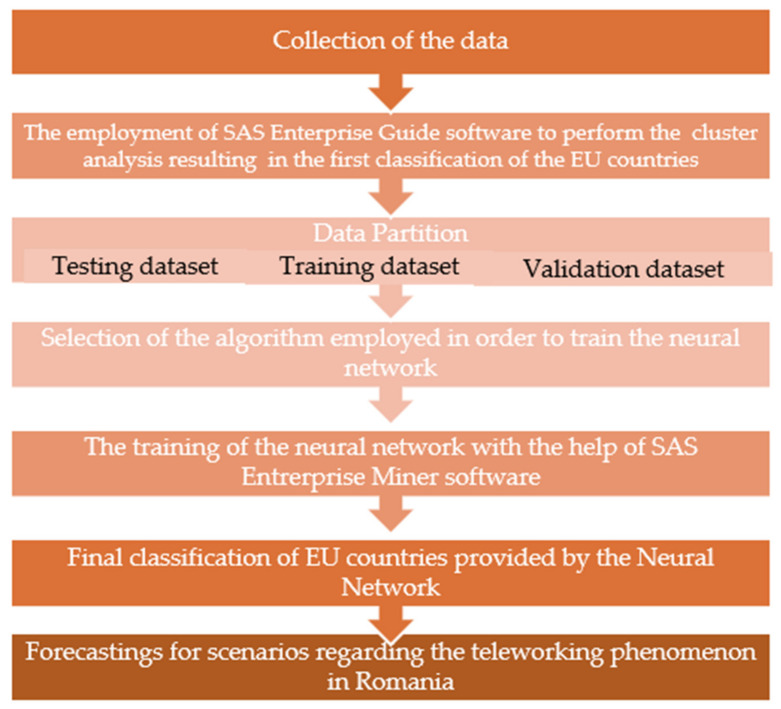
The design of the research on teleworking in EU countries and Romania.

In the light of the above theoretical grounds, the methodology employed within our study entails the following stages (Figure 4):

Stage 1: Collection of data. Based on a European-level questionnaire on various aspects of telework, we have defined ten distinctive variables regarding the level of home-based work adoption during the COVID 19 pandemic crisis. These variables were expressed as percentages in order to facilitate the comparison procedure between the data gathered from different EU countries.

Stage 2: Using the SAS Enterprise Guide software in order to acquire the initial classification of EU countries. The data matrix containing the values recorded for each variable and every single country was processed by using the SAS Enterprise Guide, version 8.3. 

In order to emphasize the similitudes and the discrepancies between EU countries (in respect to their willingness to adopt teleworking) through the medium of a systematic analysis of ten relevant variables, in this preliminary stage, we have employed the hierarchical cluster method. In this case, the algorithm that was applied was adapted to the process of building the tree cluster structure (i.e., the dendogram). 

Thus, Ward’s method was chosen in order to characterize the similitudes and the discrepancies highlighted between objects that form different clusters [43]. The method involves the minimization of the error sum of squares (also known as inertia) for the objects in the clusters, the error being calculated as the distance between an object and the center of the class it belongs to. 

Thus, the hierarchical clustering approach led us, after successive calculations of error sum of squares, to a first classification of countries into three different groups that differ significantly relative to the main issue addressed by our research but at the same time, gather homogeneous elements within each group. The output variable defined in this manner reflected the membership of each country to one class or another, and these classes were symbolized by Class1, Class2, or Class3. The neural network developed in Stage 4 was aimed at improving the first taxonomy generated by the cluster analysis by minimizing the probability of including a country into an inappropriate class.

Stage 3: The individualization of three datasets. As required by the neural network training process, we divided the input data into three categories: training data, validation data, and test data. The percentages for each type of data were to be estimated the moment that the model was defined: 60% from the input data used to train the neural network, 20% was used to validate the results, and 20% was used to test the neural network after training.

Stage 4: The training of the neural network. The neural network employed in order to classify the EU countries according to their willingness to embrace teleworking was a *multilayer perceptron network* using a group of ten variables on remote work (denoted by I1-I10) and an intermediate layer with six neurons as input data. The neural network was trained with the help of SAS Enterprise Miner, version 15.2.

The activation functions for the neural network were defined by the following formulae [44]:

(1) $$ClassZ=\alpha_{0Z}+\overset{6}{\underset{i=1}{\sum }}\alpha_{i}*H_{i}\;\;,\;Z=\bar{1,3}\;and\;\alpha_{0Z}\;is\;the\;bias$$

(2) $$H_{i}=w_{i0}+\overset{10}{\underset{j=1}{\sum }}w_{ij}*Ij\;,\;i=\bar{1,6}\;and\;w_{i0}\;is\;the\;bias$$

The activation function belongs to the hyperbolic tangent type, and it was applied to the input-to-hidden layer weights, which had and exponential distribution that was in a range between [−1, 1]. The formula applied for the activation function is presented below [45]:

(3) $$f\left(x\right)=\frac{e^{x}-e^{-x}}{e^{x}+e^{-x}}$$

The rationale behind choosing the hyperbolic tangent function for our model was related to the fact that it leads to faster convergence in the optimization process, as compared to various logistic functions.

Because we only used one output variable—the assignment to one class of countries or another—there was no need for several hidden layers to be placed in the architecture of the neural network. The number of neurons in the hidden layer was determined experimentally, the appropriate results being obtained for the six-neuron alternative. 

We chose the Levenberg–Marquardt algorithm to train the neural network because of its stability, celerity, and high level of convergence. The technique aims at minimizing the sum of the squared errors of the hyperbolic tangent function (Tanh) and incorporates both the steepest descendent algorithms and the Gauss–Newton method, as depicted in Equation (4) [44]:

(4) $$w^{\left(n\right)}=w^{\left(n-1\right)}-{\left(J^{\left(n\right)t}*J^{\left(n\right)}+A^{\left(n\right)}*I\right)}^{-1}*J^{\left(n\right)t}*r^{\left(n\right)}$$

where:

*J*^(*n*)^ is the Jacobian matrix of first derivations of the error function with respect to each weight estimate;

*A*^(*n*)^ is the second derivative of the residual squared sums of the parameter estimates at the *n*th iteration;

*r*^(*n*)^ is the vector of the residuals at the *n*th iteration.

Stage 5: Final classification of EU countries provided by the neural network. 

From the literature, we know that neural networks have the potential to indicate object membership to a pertinent class more accurately then any of the traditional hierarchical clustering methods [46]; the completion of this stage was aimed at improving the precedent systematization acquired in Stage 2. 

In order to ensure the learning process, the neural network needs to be provided with several patterns, which arise from a previous classification. This previous systematization may be instituted either in an empirical manner or as the result of another analysis—in our case, cluster analysis. 

By showing the number of classes that could be highlighted in any particular case, we assume a certain level of classification error. Through the training of the neural network, we seek to minimize this error by reaching a categorization that is as close as possible to the real situation while providing the values of the network transfer functions. 

Consequently, a future classification would not imply additional research efforts in order to train the network, but the values associated with a specific country that did not take part in the initial training process could simply be added, and the country would be assigned to the proper group by taking the transfer functions into consideration. 

In the specific case of the neural network developed in our research, the number of neurons included in the input, hidden, and output layers were 10, 6, and 3, respectively. 

As described in Section 3.1, the network that generated the lowest value for the mean squared error (MSE) in the validation dataset represented the optimum choice for our research, as it ensured the prediction rigorousness for the model. The training process continued until the network accomplished the closest output value to the sought-after output by continuously modifying the unit weights correspondingly [47].

Stage 6: Following the successful training of the neural network, a few scenarios and practical implications were drawn in order to increase the level of telework adoption by Romanian employees, on the strengths of both the global tendencies highlighted in paragraph 2 and the unprecedented challenges of the pandemic.

### 3.3. Data and Measures

The answers provided by employees from various fields and that were registered between 22 June and 27 July 2020 were classified into the following categories: work–life balance, quality of work performed, work experience at a distance, and the health and safety of employees [2]. For comparative purposes, the datasets involved in the research are percentages within the range 0 to 100 and reflect the share of respondents that totally agree or agree with a specific statement/question (included in the Eurofund questionnaire).

The respective shares assess employee willingness to adopt the new legal arrangements and procedures that facilitate telework. The variables extracted from the 2020 Eurofound survey that were assumed as highly relevant from the point of view of reflecting the degree at which employees adopted telework (Table 1) were used in order to substantiate the architecture of our neural network. 

The first variable, symbolized by I1—high and very high satisfaction on the amount of work submitted—was assessed based on the answers provided by the respondents to the following statement: “I am satisfied with the amount of work I have to do”, with the answer options: totally agree (1), agree (2), indifferent (3), disagree (4), totally disagree (5), do not know/do not answer (6). The response analysis illustrated a high percentage of respondents who agreed and totally agreed with the abovementioned statement; the sums of the percentages determined for each EU country are displayed in the second column of Table 2.

The I2 variable extracted from the survey—high and very high satisfaction on the quality of work submitted—was estimated on the grounds of the statement “I am satisfied with the quality of work I have to do”, which had the same answer options as the previous question. The sum of the percentages provided by the answers “totally agree” and “agree” was also kept further among the datasets of our model.

The I3 variable—work in optimal conditions with the equipment from home—was assessed by virtue of the statement “With the equipment I had at home, I was able to carry out my work in optimal conditions”, with the same options as the previous questions that were used in the Eurofound research. The percentages provided by respondents who agreed and totally agreed with the statement are summarized in Table 2.

The variable denoted by I4—satisfaction with the experience of working from home—follows the same construction reasoning as the above, cumulating the answers agree and totally agree with the statement “Overall, I am satisfied with the experience of working from home”.

The variable symbolized by I5—work normally involves physical contact with other people most of the time, quantifies the affirmative answers to the question: “In your activity, you are in direct contact with other people (colleagues, clients, passengers, students, patients and others)?” Other possible answers to this question were the following: sometimes, rarely, never, and do not answer.

The I6 variable—maintaining constant work performance—quantifies the share of respondents who have chosen the alternative “is kept constant” to the question “During the COVID-19 pandemic, how did the performance in the submitted work change?”, other possible options for answering the abovementioned question were: increased a lot; increased to a lesser extent; decreased a lot; decreased to a large extent; do not know/do not answer. 

The I7 variable—the risk of SARS-CoV-2 transmission in the workplace quantifies the affirmative answers of the respondents with respect to their perception on the magnitude of the threat of becoming infected with the new coronavirus while accomplishing their work tasks.

The I8 variable—not accepting work from home before the pandemic—assesses the share of employees belonging to companies inside of which the organizational culture did not integrate remote work arrangements before the health crisis.

Keeping the same work schedule during the pandemic, I9, estimates the respondents’ opinions regarding the modification of their work schedule compared to status-quo before the forced adoption of remote work. Thus, the figures displayed in Table 2 revealed that, in a very large proportion of people, the same work schedule as before the outbreak of the pandemic was maintained. 

Finally, non-involvement of family issues and duties in performing work tasks, denoted by I10, shows that the majority the people who completed the questionnaire declared that family problems and responsibilities did not prevent them from successfully conducting the obligations imposed by their job.

The initial database employed in order to achieve the first classification of countries with the SAS Enterprise Guide is displayed in Table 2.

Once the variables were defined and the values recorded for each variable were collected, the next stage of our research methodology implies the establishment of the initial classification of countries by virtue of the SAS Enterprise Guide. The results are presented in the paragraph below.

## 4. Results

### 4.1. Descriptive Statistics

Before performing Ward’s algorithm in order to obtain a first accurate classification of EU countries, the SAS Enterprise Guide provided the descriptive statistics of data series materialized in the relevant measures of central tendencies and measures of variability (see Table 3 and Table 4).

We can conclude that our datasets conform to the normal distribution, as the majority of measurements concentrate around the mean value, while skewness and kurtosis fall in the acceptable range (below +1.5 and above −1.5) [48]. With respect to their vault, six of our data series are platykurtic (i.e., I2, I5, I6, I8, I9, I10), while the others are leptokurtic; form the point of view of their symmetry, I1, I2, I3, and I7 are asymmetric right, and the others are asymmetric left. 

Moreover, the values that are very closed to zero of bimodalities and that are displayed in the last column of Table 3 drove us to the conclusion that we are dealing with a unimodal distribution. 

The eigenvalues presented in Table 4 provide further detail about the amount of information introduced into the model by each variable, indicating when and whether it is necessary to give up a variable.

In the case of our research, all of the variables are important to some extent, as they introduce valuable and accurate information. Consequentially, all of the variables were kept further on in our research, contributing to the model building process of the neural network.

### 4.2. The Cluster Analysis

**Figure 5:**
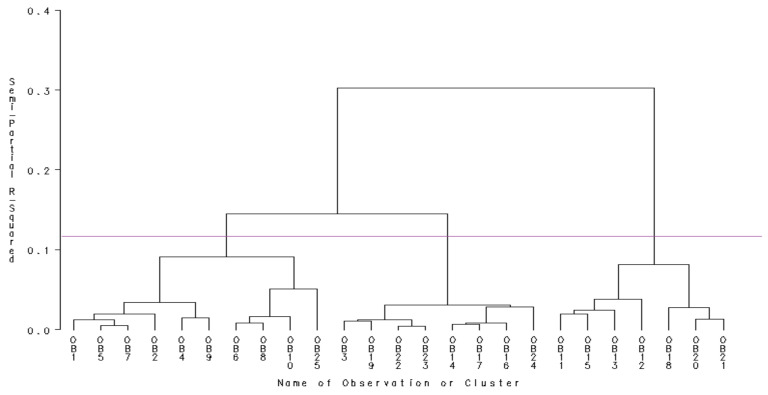
Cluster generation dendogram provided by the SAS Enterprise Guide software.

Since all of the data series were retained in our analysis, the next stage was represented by the analysis performed on the dendogram yielded from the SAS Enterprise Guide software and the consequent rationalizations undertaken in order to find the best way of grouping observations (Figure 5).

Reading the dendogram above, we could establish the number of classes the EU countries should be divided into, with the following options in mind:

- If the graph is cut below 0.1, then five classes of countries could be individualized, but the distance between them would be a very small one. Although this situation is the most accurate from the statistical point of view, it does not fit reality because the existing dissimilarities between countries in terms of telework adoption might be easily overlooked;
- If the cut is between 0.2 and 0.3, then two large and well-defined classes could be distinguished, but the differences between the objects that compose the same class would be significant. Such an approach would affect the object heterogeneity and would be inconsistent with the very theoretical foundations of cluster analysis;
- If the cut is in the range [0.1, 0.2] and is closer to 0.1, then we should obtain three distinct classes that meet the requirements of the cluster analysis both from the statistical point of view and from the real perspective of the issue being addressed.

Thus, following the first determination, the components of each group of countries are:

- Class 1: Denmark, Sweden, Finland, Germany, Ireland, the Netherlands, Luxembourg, Belgium, Italy, Austria;
- Class 2: Slovakia, France, Bulgaria, Romania, Czech Republic, Hungary, Slovenia, Estonia;
- Class 3: Greece, Croatia, Portugal, Spain, Lithuania, Poland, Latvia.

According to our research design, the next stage of our methodology consists of the further optimization of the initial systematization of EU countries provided by the cluster analysis. To this end, we focused on developing a neural network-based classification model in order to bring to the fore the main features of the EU states in terms of their openness towards telework adoption.

### 4.3. The Training of the Neural Network

As far as the role assigned to each variable in the NN model is concerned, we made the following assumptions:

✓ The values of variables I1–I10 fall into the input dataset category;
✓ the output variables Class 1, Class 2, Class 3 were considered categorical variables with two possible values: 1 (if a specific country belongs to that class) and 0 (if the country does not belong to that class).

We employed the Levenberg–Marquardt algorithm, which involved both the data matrix with the values recorded for each variable and each EU country and the data regarding the differentiation of the initial classes provided by the SAS Enterprise Guide program. 

Empirical studies have indicated that neural networks are susceptible to overfit the data in the training set, which is the cause of producing faulty out-of-sample predictions. In order to avoid this pitfall, we used a procedure called early stopping, which implies the partition of the dataset into three components: the training data set; the testing dataset; and the validation dataset. 

The training dataset was applied in order to determine the network weights, while the testing dataset was laid aside for out-of-sample prognosis. Finally, the validation dataset represents a data partition that is not employed in the training process of the neural network, but it functions as barometer of the out-of-sample prediction accuracy associated with the network [49].

On that basis, we divided the datasets from Table 2 as follows:

- The *NN training dataset*, covered by data gathered for Denmark, Finland, Germany, the Netherlands, Greece, Croatia, Portugal, France, Spain, Romania, Lithuania, Czech Republic, Poland, Latvia, and Austria;
- The *NN validation dataset*, represented by data collected for Sweden, Ireland, Luxembourg, Belgium, and Slovakia;
- The *NN testing dataset*, encompassing data put together for Slovakia, Italy, Bulgaria, Hungary, and Estonia.

**Figure 6:**
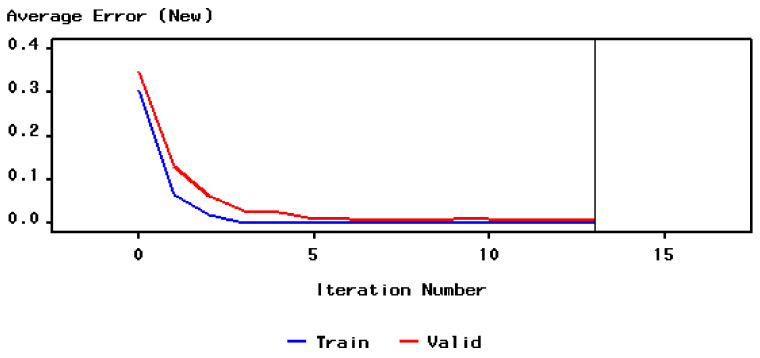
The evolution of both the training error and the validation error.

Before applying the chosen algorithm to the neural network, the values of the specific indicators were determined (see Figure 6). Accordingly, the value of the objective function was 0.304.

**Figure 7:**
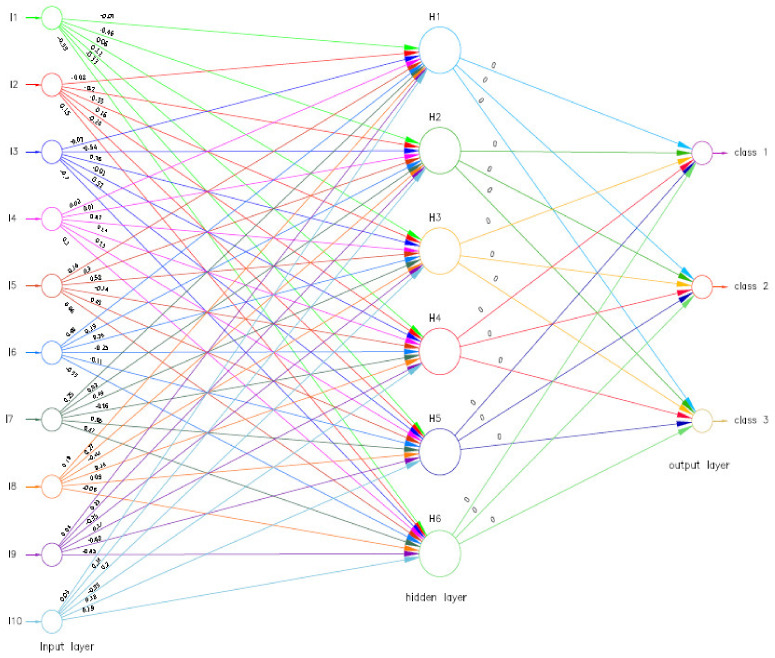
The neural network associated with the classification model (before training).

For the process of training the neural network, 13 iterations were necessary until the expected result was reached. The evolution of the average training and validation errors are presented in Figure 7. If in the first two iterations, the values of the two types of errors are high; starting from the fifth iteration, they begin to decrease, approaching the zero value. The training procedure stopped when the prediction error reached the minimum value both in the validation and the training datasets. 

**Figure 8:**
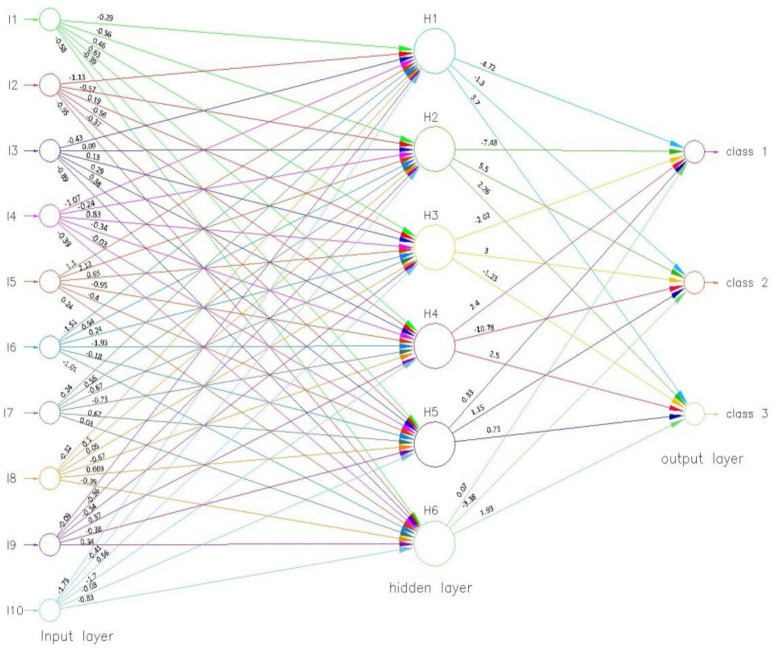
The neural network associated with the classification model (after training).

In the process of changing the weights with very single iteration, the neural network is considered to be learning. Following the successful training of the neural network, the values of the specific indicators were estimated as follows (see Figure 8). Accordingly, the value of the objective function was 0.000014.

For the future use of the neural network presented above, the transfer functions are as follows:

- For the intermediate layer, the functions are presented in Equations (5)–(10):
H1 = −0.29 × I1 − 1.13 × I2 − 0.43 × I3 − 1.07 × I4 + 1.1 × I5 − 1.52 × I6 + 0.24 × I7 − 0.32 × I8 − 0.09 × I9 − 1.75 × I10; H1 = −0.26 + H1;(5)H2 = −0.56 × I1 − 0.57 × I2 + 0.08 × I3 − 0.24 × I4 + 2.12 × I5 + 0.94 × I6 + 0.55 × I7 + I8 − 0.36 × I9 − 0.41 × I10; H2 = 0.54 + H2;(6)H3 = 0.46 × I1 + 0.19 × I2 + 0.13 × I3 + 0.83 × I4 + 0.65 × I5 + 0.24 × I6 − 0.67 × I7 + 0.05 × I8 − 0.54 × I9 + 0.56 × I10; H3 = 1.13 + H3;(7)H4 = 0.63 × I1 − 0.56 × I2 + 0.29 × I3 − 0.34 × I4 − 0.95 × I5 − 1.93 × I6 − 0.73 × I7 − 0.67 × I8 + 0.37 × I9 − 1.7 × I10; H4 = 1.88 + H4;(8)H5 = − 0.39 × I1 − 0.37 × I2 + 0.38 × I3 − 0.032 × I4 − 0.4 × I5 − 0.18 × I6 + 0.67 × I7 + 0.003 × I8 − 0.38 × I9 − 0.03 × I10; H5 = −1.12 + H5;(9)H6 = −0.58 × I1 − 0.55 × I2 − 0.89 × I3 − 0.39 × I4 + 0.24 × I5 − 1.01 × I6 + 0.031 × I7 − 0.39 × I8 + 0.34 × I9 − 0.83 × I10; H6 = −0.02 + H6;(10)
- *for the output layer,* the functions are described with the help of Equations (11)–(13):
Class1 = −4.72 × H1 − 7.45 × H2 − 2.02 × H3 + 2.41 × H4 + 0.33 × H5 + 0.073 × H6; Class1 = −2.12 + Class1;(11)Class2 = −1.3 × H1 + 5.51 × H2 + 3 × H3 − 10.78 × H4 + 1.145 × H5 − 3.38 × H6; Class2 = −1.03 + Class2;(12)Class3 = 5.71 × H1 + 2.26 × H2 − 1.23 × H3 + 2.75 × H4 + 0.73 × H5 + 1.93 × H6; Class3 = −1.63 + Class3.(13)

As we stated above, after the successful training of the neural network, we had to shift the focus to the testing dataset, i.e., those data which were not involved in the operational process of building the neural network but that served as verification instruments for the outcomes generated by the model. Therefore, we checked whether or not the countries maintained the class to which they were initially distributed the moment we employed the NN testing dataset. Table 5 shows the results that were achieved using the above-mentioned approach. 

According to data displayed in Table 5, Slovakia, Bulgaria, and Hungary remain in Class 2 when employing the testing dataset, while Italy moves to Class 3, and Estonia relocates to Class 1. Finally, the NN model yields the following class configuration:

- Class 1: Denmark, Sweden, Finland, Germany, Ireland, the Netherlands, Luxembourg, Belgium, Estonia, Austria;
- Class 2: Slovakia, France, Bulgaria, Romania, Czech Republic, Hungary, Slovenia;
- Class 3: Greece, Croatia, Portugal, Spain, Lithuania, Poland, Latvia, Italy.

## 5. Discussion

### 5.1. General Overview on the Degree of Telework Embracement by Groups of EU Countries

In spite of the tremendous technological developments that facilitated the occurrence of mobile and smart telework, inertia occurred in the pre-pandemic period, which mostly derived from inflexible work cultures and low/moderate levels of concern exhibited both by some employers and employees who were reticent in acquiring modern equipment and in putting together management tools to implement the new work arrangements [50].

The datasets employed by the present research and its outcomes are consistent with other recent empirical studies from the literature that indicate that the COVID-19 health crisis turned out to be an exceptional force in the speeding up the trend of telework adoption across EU countries [3,4,51,52,53,54]. Although emergency actions, which were put into operation in order to avoid SARS-CoV-2 proliferation among employees, were able to bring about a long-expected shift in terms of the settling down of ”working-from-home practices”, a set of drivers responsible for the degree of telework adoption were mentioned both by the pre- and post-pandemic literature [3,4,8,16,20,30,54]. While there is a consensus in relation to the main determinants that impact the relationship between telework and organizational performance—such as the adjustability of work arangements; employee degree of self determination in conducting their work tasks; incresed protection against ilnesses; the diminishing trend of the operational costs of enterprises; the employment of appropriate communication tools; efficient organization of business operations, etc.—the assessement of their consequences and importance varies to a large extent from one author to another [55,56].

On the basis of bringing together these research efforts and the outcomes released by the 2020 Eurofund research on living and working conditions, we were able to differentiate three basic groups of European countries with relatively homogenuous pecularities on the issue of teleworking. Such pecularities, together with their triggering mechanisms, constitue the focus of the discussions detailed within this paragraph. 

Thus, the first class of countries illustrated a level of satisfaction regarding the experience of working from home, which was higher the European average. This was due to the fact that Finland, Denmark, Sweden, and the Netherlands are considered to be trendsetters at the EU level in terms of digital performance [57]. Hence, on the 1st October 2020, the governing bodies from the Nordic and Baltic European countries consented to a joint Ministerial Declaration—Digital North 2.0—with ambitious plans in terms of becoming the preeminent sustainable and integrated region globally by 2030 through the development and employment of innovative technologies in order to face staggering societal transformations, such as pandemics, green revolution, or teleworking [52]. Under the circumstances, employees from the countries included in Class1 (Denmark, Sweden, Finland, Germany, Ireland, the Netherlands, Luxemburg, Belgium, Austria) have used, to a great extent, the equipment they possessed at their residence; therefore, no additional time was needed for them to take part in training programs or to get used to new technologies. Consequently, within the above-mentioned countries, the level of personal satisfaction with the amount of effort undertaken and the satisfaction of the quality of the work performed were above the EU average. 

The experience and knowledge accumulated on home-based teleworking before the outbreak of COVID-19 pandemic in the countries included in Class1 appeared to have played a crucial role in their degree of telework adoption. Thus, the significant jump in the number of employees who started teleworking during the crisis demonstrated that, with the availability of adequate technology, communication instruments, and managerial restructuring strategies, significantly more jobs became “teleworkable” than previously estimated [11]. Due to the fact that working-from-home procedures had been accepted before the pandemic in this group of countries, the foundations for the new work arrangements (rather low contact with other people, small-level risk of SARS-CoV-2 contamination, high degree of digitization, important job flexibility, etc.) were already met; therefore, work performance could be maintained at a constant level, while employees learned how to manage a pre-established schedule so that they could be able to successfully combine family problems and responsibilities with work tasks. This conclusion is in line with other recent empirical studies that demonstrate that the general increase in the degree of teleworker satisfaction raises the amplitude of their job commitment [58,59,60].

The second class is made up of the following countries: Slovakia, France, Bulgaria, Romania, Czech Republic, Hungary, and Slovenia. In these states, the employee flexibility with regard to the new working conditions during the pandemic was slightly different compared to the previous group. First, there was not a high prevalence of teleworking in the pre-COVID era (as described in paragraph 2), which made the mutation towards “the forced telework mode” more challenging, following sinusoidal trajectories in some cases [12]. Employees needed various work equipment they did not have in their possession at the moment that lockdowns began, so they required a certain number of additional training hours to bridge their ability gaps [11]. Consequentially, their level of satisfaction towards the quality and quantity of work submitted descended around the average values registered by the sample under analysis. On the other hand, the risk of SARS-CoV-2 infection was estimated as being higher in comparison with the level of the threat assessed by the employees from the previous group; such circumstances can be correlated with the fact that within this class of countries, teleworking from home before the pandemic outbreak was not widely accepted and that the activities normally carried out at work involved contact with other people to a large extent. 

Nevertheless, within this group of EU states, the same work schedule as in the pre-pandemic period was broadly maintained, and the work performance remained fairly steady. Thus, recent studies from the literature have shown that results-based management through which both supervisors and employees comply with a conventional productivity assessment system represents the most likely approach to ensure maximum organizational efficiency. Such work procedures lie with four basic planning components: the establishing of objectives; the coherent division of tasks between teleworkers; a clear reporting system on outcomes; and a high level of autonomy given to employees in order to coordinate their own work [11]. Furthermore, it seems that within this group of countries, the high level of flexibility of the work submitted was responsible for the relative amount of employee success in terms of balancing their professional and personal lives [61]. 

The third class was composed of the following countries: Greece, Croatia, Portugal, Spain, Lithuania, Poland, Latvia, and Italy. Among this group, we see those states which were seriously shaken by the pandemic onset (such as Italy, Spain, or Portugal) and who had registered low levels of pre-crisis telework prevalence (see paragraph 2). Therefore, their complex and sudden shift to remote work procedures due to the strict safety measures implemented in relation to COVID-19 was both unprecedented and brutal. We can easily understand why the rapid adoption of work-from-home practices brought about various reasons for dissatisfaction from employees belonging to this group of countries: the lack of suitable equipment; the failure to comply with the same work schedule; the absence of clear demarcations between working hours and personal life; the low level of organizational readiness that significantly impacted employee performances and their sense of professional commitment, etc. [62].

However, a few positive effects of teleworking were highlighted in the recent literature in the cases of employees originating from Italy, Spain, Portugal, or Lithuania: important reductions in commuting time and costs; substantial raises in wages brought about by relevant increases in the number of working hours submitted; improvements in worker digital and soft skills; the opportunity to promote a healthier lifestyle; the higher flexibility in organizing work-from-home tasks; enhanced levels of job satisfaction and motivation, etc. [51,63,64]. 

Moreover, the 2021 version of the Eurofound online research study demonstrated that even with the moderation of lockdown procedures and the incremental re-starting of economic activities in mid-2020, a significant number of European employees (over three-quarters) confirmed their willingness to continue to work remotely, at least within a partial regime. These data show that for the countries included in the third class, the early inherent difficulties in adopting the new work arrangements were left behind to a large extent, and telework practices tended to manifest themselves as a substantially more widespread system than before the occurrence of the COVID-19 health crisis [62].

### 5.2. Drawing Up a Few Scenarios in Order to Increase the Degree of Telework Adoption in Romania

Unlike other countries from the EU that largely acknowledged the concept of telework, Romania only formalized this new work model as of April 2018, when many regulations addressing the remote work phenomenon were promulgated. Prior to these procedures coming into force, remote work was implemented in an ad hoc manner, according to exigencies imposed by the companies involved; under the circumstances, it is obvious that the possibilities of exploiting the enormous potential of home-based working were neglected to a great extent. Consequently, our study attempts to fill a gap in the Romanian literature, which, until quite recently, was excessively concerned on labor judicial subjects [54].

Despite an abrupt growth in the share of teleworkers during the COVID-19 crisis, Eurostat data show that Romania still lacks substantial adjustments in order to overcame the main obstacles that currently obstruct an upsurge in telework expansion: the predominance of handiwork and low-qualified employees on the labor market; the small share of personnel employed in ICTS and related fields; incomplete development of digital skills and infrastructures (except for the knowledge intensive industries); the incompatibility between the domestic conventional managerial style and the remote work pre-requisites; the inherent limitations imposed by phenomenon of overcrowding in homes on the quality and quantity of work submitted, etc. [65,66]. 

However, some recent sector-level surveys undertaken in Romania during 2020 showed the emergence of pioneering domains in terms of teleworking, such as the business service area, the financial sector, or the public administration. For instance, half of the companies from the business service sector that took part in Price Waterhouse Cooper’s questionnaire implemented full-time telework, while other 45% called on hybrid work patterns. Moreover, 74% of companies from the financial sector put telework into practice, either part time or full time. As far as the public sector is concerned, another survey performed by the National Institute for Administration (2020) revealed that over 50% of management and executive personnel worked remotely and that about 30% used other mixed work procedures. On the other hand, other less-teleworkable occupations such as those in logistics, manufacturing, and the pharmaceutical industry registered shares of teleworkers below the national average (30.1%) [65,67].

If we correlate the review of the literature with the answers provided to the 2020 Eurofound questionnaire by Romanian employees, the first conclusion would be that the country has adapted to the peculiarities of remote work to a moderate extent due to the emergency measures imposed by the necessity of avoiding workplace contamination with the SARS-CoV-2 virus. Although the subject is still under-researched, the willingness to continue teleworking in the post-COVID era seems to be strongly correlated with the quality of the experience that teleworkers underwent amidst the lockdown phase [68].

In that view, on the grounds of the neural network developed in paragraph 4.3, we were interested in drawing up a few feasible scenarios that could indicate adjustments to be made for Romania in order to access Class1 in terms of telework compliance. Three main scenarios were generated following the employment of the above-mentioned statistical model and the determination of the network transfer functions (Table 6).

The first scenario recommends some changes that can be made in the values of input variables I3, I5, and I9:

- I3 > 75%, meaning that teleworkers should be able to procure their own suitable equipment that will allow them to conduct their work in optimal conditions. Although the Telework Law that came into force in Romania in 2018 stipulated the employers’ responsibility to provide remote employees with the necessary work equipment, the legal provisions were not fully observed due to the emergency circumstances. However, through Governmental Emergency Ordinance no. 132/2020, employers were granted a one-off payment of 2500 lei (around EUR 514) for each employee who worked from home for at least 15 days. In order to support the post-pandemic increase in the uptake of telework according to Scenarios 1 and 2, governmental authorities have to continue the development of funding programmes to facilitate access for low-income employees to proper infrastructures and tools that are adequate for teleworking;
- I5 < 45%, meaning a substantial decrease in the amount of physical contact with other people while performing work tasks. To this end, modern communication channels must be enhanced in order to establish efficient connections between employees: emails, phone calls, text messages, video conferences, social media sites, other dedicated platforms, etc.;
- I9 > 45%, i.e., the level of maintaining the same work schedule also has to be increased in order to establish clear boundaries between professional life and family responsibilities in the framework of teleworking. Thus, research has revealed that preserving the number of working hours while gaining additional flexibility in organizing the working schedule is synonymous with raising the degree of employee job satisfaction [54,69].

The second scenario takes into account adjustments that could be implemented in the following input variables:

- I10 > 80%, which is to say that the degree of the non-involvement of family problems and responsibilities in the process of conducting work tasks represents an issue that requires greater importance. Thus, recent studies from the literature have suggested that the burnout generated by constant connection to work tasks, supplementary demands, unreasonable deadlines, and the lack of group-problem solving in the workplace are prone to impair teleworker satisfaction. On the other hand, a positive relationship was established between high levels of professional competences, work–life balance, organizational culture degree of openness towards remote work, and overall well-being [70,71]. Moreover, safety issues in the framework of telework must be addressed both at the company and at the national level to serve the need for new legislative measures regarding employee protection against the psychosocial risks that can occur due to rapid digitalization;
- I6 > 45%, which reflects the focus on maintaining or increasing work performance—a complex variable—which can simultaneously be affected by a few relevant determinants such as individual characteristics (i.e., self-management procedures), home environment factors (i.e., the existence of suitable telework pre-requisites), and job peculiarities (i.e., balanced workload) [66].

The third scenario focus the attention on measures that could adjust the following input variables:

- I8 > 75%, meaning that in the post pandemic era, public authorities and companies have to stress the importance of raising the employee level of awareness regarding telework adoption [72]. Moreover, at the company level, multinationals from the EU countries have instituted company-characteristic teleworking procedures, often pushed forward by the precipitated exposure to remote working during the emerging state. Thus, recent qualitative research involving main Romanian stakeholders [65] revealed that the measures implemented in the emergency state both at the public and private levels were defficient and rather unsyncronized. Within this framework, the need for further investment in digital infrastructures, re-skilling programmes, and training courses on well-being has been intensely exposed;
- I9 > 45% and I10 > 75%, that is to say additional far-reaching reforms need to be made in terms of telework legislation (especially for the public domain), with direct implications for the arrangements regarding the improvement of work–life balance. For instance, Romania should follow the example of other Member States (such as France, Germany, Spain) that adopted specific legislation in order to restrict out-of-working hours electronic communications and to defend the right to disconnect (that protects the employee against the psychosocial stress caused by any unreasonable requirements regarding his/her permanent availability on line). On another train of thought, at the European level, two recent directives—*The Work–Life Balance Directive* and the *Transparent and Predictable Working Conditions Directive*—are due to be fully applied by the EU countries by 2022 and address, inter alia, the protection of flexible work schedules for employees with children up to 8 years of age while implementing well established working time patterns [62].

Besides the aspects highlighted above, alternative scenarios to strengthen the growth of post pandemic telework adaptability in Romania can be developed based on the operation of the neural network. Nevertheless, in order to judge the scenarios generated by the model as feasible, one must pay attention to their degree of compliance with the existing legislative framework, the work culture, and peculiarities of domestic management strategies.

## 6. Conclusions

Against the available background of a very short timespan in order to bring to the light the main determinants that resulted in the rapid adoption of telework by EU countries, our paper explored the similarities and discrepancies between the Member States in their effort to adhere to the new work arrangements instituted by the pandemic situation. The methodology employed within our research enabled us to classify the examined countries according to ten relevant variables related to various aspects such as the availability of work equipment; the degree of satisfaction with the experience of working from home; the risks related to potential contamination with SARS-CoV-2 virus; the employee openness to adhere to working-from-home patterns; the possibility of maintaining work–life balance objectives while teleworking; the level of satisfaction on the amount and the quality of work submitted, etc. The results of our research demonstrated the existence of significant disparities in terms of telework adaptability, such as low to moderate levels of adaptability (exhibited by countries such as Greece, Croatia, Portugal, Spain, Lithuania, Latvia, Poland and Italy); fair levels of adaptability (encountered in Slovakia, France, Bulgaria, Romania, Czech Republic, Hungary, and Slovenia), and high levels of adaptability (shown by highly digitalized economies such as Denmark, Sweden, Finland, Germany, Ireland, the Netherlands, Luxembourg, Belgium, Estonia, and Austria). As far as Romania is concerned, several scenarios regarding the future development of telework were discussed, together with a set of policy indications in order to improve the necessary endowments and to substantially reform the legislative framework according to these new realities. 

The contribution of the present paper to the existing literature can be put forward in several ways. First, it offers a comprehensive review of the concepts related to telework and the artificial neural networks applied in the field of economic sciences. Secondly, the paper represents, to the best of our knowledge, the first attempt at investigating the degree at which telework has been adopted within the framework of COVID-19 pandemic by means of modeling employee perceptions utilizing neural networks. Thirdly, the study shed new light in respect to the scenarios that should be applied in Romania in order to facilitate a significant increase in the share of remote employees.

On the other hand, the authors are aware that the present research is not free of limitations. First, neural network training requires a large volume of data and can suffer from overtraining, but this drawback can be mitigated if a training dataset is properly used and if the authors are able to choose a suitable architecture that best fits the problem to be solved. 

Secondly, as far as the databases are concerned, it is acknowledged that official EU-wide statistics on teleworking during COVID-19 the pandemic are still not available; however, the unprecedented pace of transformations brought about in the labor market by the health crisis require additional research efforts towards a better understanding of the determinants that facilitate the shift from traditional working patterns to new ones. Under the circumstances, using preliminary data extracted from the 2020 Eurofound survey on living, working and COVID-19 constituted the best possible choice for us. Thus, an in-depth analysis of the telework phenomenon by using the most recent datasets published by Eurofound [73,74] with the employment of both discriminant analysis and a neural network model represents an interesting avenue of research that is worth further exploration. 

## Figures and Tables

**Table 1:** Input variables incorporated in the neural network architecture.

Crt. No	Symbol	Variable Name
1	I1	Satisfaction on the amount of work submitted
2	I2	Satisfaction on the quality of work submitted
3	I3	Work in optimal conditions with the equipment from home
4	I4	Satisfaction with the experience of working from home
5	I5	Physical contact with other people during working
6	I6	Maintaining constant work performance
7	I7	The risk of SARS-CoV-2 transmission in the workplace
8	I8	Not accepting work from home before the pandemic
9	I9	Keeping the same work schedule during the pandemic
10	I10	Non-involvement of family issued and duties in performing work tasks

Source: Authors’ elaboration from the 2020 Eurofund survey Living, working and COVID-19: Round 2—July 2020.

**Table 2:** Percentages of telework adoption associated with the study variables.

	I1 (%)	I2 (%)	I3 (%)	I4 (%)	I5 (%)	I6 (%)	I7 (%)	I8 (%)	I9 (%)	I10 (%)
Denmark	55	74.9	67.1	66.9	48.8	32.4	26.8	54.5	56.8	80.7
Sweden	53.5	63.2	65.2	60	42	33.7	43.2	65.6	48.3	77.8
Slovakia	53.7	68.7	67.3	61.1	75.3	37.2	32.5	70.4	43.3	71.6
Finland	64.6	71.2	78.6	70.8	52.9	43.1	29.4	52.5	44.2	70.2
Germany	53.3	69	70.4	58.9	50.6	36.2	29.8	58.2	44.5	74.1
Ireland	57	69.7	59.2	59.6	46	26	35.2	69.4	34.4	67.4
Netherlands	56.3	69.8	72	60.2	43	36	30.8	61.7	50	79.6
Luxembourg	57.9	68	69.2	67.1	47.2	27	42	64.8	32.1	68.1
Belgium	57.1	69.3	71.6	62.1	44.1	37.1	36.1	54.4	39.2	63.3
Italy	54.6	60.3	65.1	55.9	56.4	21.1	31.5	73.1	29.4	68.5
Greece	47.7	56.6	53	44.5	72.1	26.2	60.2	63	31.6	68.8
Croatia	46.9	56.9	60.2	48.4	71.5	36.1	63	75.2	50.4	55.1
Portugal	51.5	63	67.7	56.3	67.6	28.6	71	63.7	25.1	57.4
France	48.5	63.9	65.9	59.3	65	36.5	46	69.1	32.9	73.1
Spain	50.6	58.8	59.1	52.8	59.9	29	51.1	72.2	26.5	58.3
Bulgaria	53.6	63.6	66.1	62.6	61.1	39.1	44.2	66.8	45.1	73.4
Romania	55.8	70.8	65.2	60.7	67.5	40	49.9	70.5	37.6	67.6
Lithuania	47.8	56.6	60.9	57.4	73.8	29.5	29.9	67.3	42.1	70.7
Czech Rep.	61	72.2	75.8	68.2	75.6	41.5	36.9	61.2	43.2	72.3
Poland	44.6	58.7	63.5	39.1	65.7	33	36.9	62.8	31	58.7
Latvia	41.3	58.9	63.5	47.7	61.7	37.7	44.4	62.8	43.5	65
Hungary	58.3	72.7	65.9	64.2	67	38.1	42	64.7	41.6	83
Slovenia	56.5	71.4	66.5	61.5	70.6	41.6	40.9	64	43.7	74.8
Estonia	56.8	63.9	68.1	64.9	61.3	34	47.8	50.4	50.5	74.2
Austria	70.6	82.1	79	72.4	56.2	34	44.6	68.2	29.8	70.6

Source: Authors’ elaboration from the 2020 Eurofound survey Living, working and COVID-19: Round 2—July 2020.

**Table 3:** Descriptive statistics for the main research variables.

Variable	Mean	Standard Deviation	Skeweness	Kurtosis	Bimodality
I1	54.1800	6.2571	0.3524	1.1521	0.2461
I3	66.6440	5.9790	0.1697	0.6238	0.2547
I2	66.1680	6.5783	0.2700	−0.2644	0.3405
I5	60.1160	10.8053	−0.2608	−1.2174	0.4860
I8	64.2600	6.4634	−0.5235	−0.1944	0.3956
I10	69.7720	7.2389	−0.3391	−0.2078	0.3477
I7	41.8440	11.1272	0.9636	0.7904	0.4586
I9	39.8720	8.4407	−0.0495	−0.8348	0.3885
I6	34.1880	5.5756	−0.5454	−0.2421	0.4089
I4	59.3040	7.9954	−0.7928	0.6568	0.4000

**Table 4:** Eigenvalues of the covariance matrix.

Variable	Eigenvalue	Difference	Proportion	Cumulative
I1	274.735896	172.544182	0.4437	0.4437
I3	102.191714	8.478875	0.1650	0.6088
I2	93.712839	33.198663	0.1513	0.7601
I5	60.514176	25.968228	0.0977	0.8578
I8	34.545948	11.356947	0.0558	0.9136
I10	23.189001	9.710345	0.0375	0.9511
I7	13.478656	5.199626	0.0218	0.9728
I9	8.279031	3.336893	0.0134	0.9862
I6	4.942137	1.351202	0.0080	0.9942
I4	3.590936	-	0.0058	1.0000

**Table 5:** Class probabilities for countries included in the testing dataset.

Country	Probability of Falling in Class 1	Probability of Not Falling in Class 1	Probability of Falling in Class 2	Probability of Not Falling in Class 2	Probability of Falling in Class 3	Probability of Not Falling in Class 3
Slovakia	0.000066711	0.999933288	0.999999997	2.475929 × 10^−9^	0.000020891	0.99997911
Italy	0.704275705	0.295724294	2.930769 × 10^−9^	0.999999997	0.994797623	0.005202376
Bulgaria	0.000580391	0.999419609	0.99999998	2.001788 × 10^−8^	0.000013891	0.999986109
Hungary	0.010475582	0.989524417	0.999999984	1.535445 × 10^−8^	1.755392 × 10^−6^	0.999998245
Estonia	0.999955401	0.000044598	3.885994 × 10^−7^	0.999999611	0.000352685	0.999647315

**Table 6:** Scenarios drawn up in order to raise the share of teleworkers in Romania.

No. crt.	The Initial Values of the Input Variables	New Thresholds for the Input Variables
1	I3 = 65.2%, I5 = 67.5%, I9 = 37.6%	I3 > 75%, I5 < 45%, I9 > 45%
2	I10 = 67.6%, I3 = 65.2%, I6 = 40%	I10 > 80%, I3 > 80%,I6 > 45%
3	I8 = 70.5%, I9 = 37.6%, I10 = 67.6%	I8 > 75%, I9 > 50%, I10 > 75%

## Data Availability

Data supporting reporting results can be found at: https://www.eurofound.europa.eu/data/covid-19 (accessed on 3 October 2021).

## Appendix A

**Table A1:** Experience of telework pre-COVID crisis (index 0-100) in each of the categories.

	Essential	Teleworkable	Partly Active	Mostly Non-Essent	Closed	All Sectors
DE	6.45	15.26	6.03	5.75	9.51	8.58
FR	12.35	19.86	11.13	9.10	14.71	13.94
IT	2.87	8.56	3.85	2.05	4.05	4.31
ES	4.02	12.11	4.14	4.08	4.04	6.01
PL	11.64	15.14	5.94	4.06	9.11	9.44
NL	20.28	41.70	15.59	19.51	21.98	25.41
RO	0.47	1.02	0.57	0.46	0.60	0.57
CZ	3.68	12.96	6.08	3.29	14.89	6.96
SE	12.11	30.59	18.71	16.26	19.27	20.56
BE	10.35	24.25	10.47	10.91	17.49	14.88
HU	2.61	7.94	4.12	2.31	5.07	4.30
AT	18.04	25.97	10.58	8.08	16.23	16.21
GR	1.70	9.05	1.29	2.48	1.61	3.59
PT	2.65	17.03	1.86	0.00	0.00	5.14
BG	0.34	1.34	0.38	0.24	0.97	0.59
FI	16.32	37.23	17.55	16.03	23.20	22.25
SK	5.20	10.96	7.13	3.74	5.70	6.42
DK	8.13	23.38	6.92	4.03	0.61	10.53
IE	7.89	13.69	0.75	2.63	0.00	6.55
HR	3.18	7.80	2.88	7.76	3.31	4.16
LT	5.24	3.64	3.48	2.07	3.72	3.70
SI	9.05	24.71	10.09	5.99	12.34	12.43
LV	5.67	5.16	2.30	1.18	4.71	3.91
EE	9.50	23.23	12.41	8.95	14.87	13.86
CY	1.41	2.82	0.73	0.69	2.83	1.78
LU	16.32	23.62	18.41	21.06	18.65	20.74
MT	3.45	13.42	4.71	5.27	9.59	7.85
UK	11.58	20.94	10.03	13.05	12.35	14.52
EU28	8.36	17.49	7.40	6.40	9.78	10.23

Source: [12].
